# Transcriptome and Differential Methylation Integration Analysis Identified Important Differential Methylation Annotation Genes and Functional Epigenetic Modules Related to Vitiligo

**DOI:** 10.3389/fimmu.2021.587440

**Published:** 2021-03-10

**Authors:** Yihuan Pu, Xuenuo Chen, Yangmei Chen, Lingzhao Zhang, Jiayi Chen, Yujie Zhang, Xinyi Shao, Jin Chen

**Affiliations:** ^1^Department of Dermatology, The First Affiliated Hospital of Chongqing Medical University, Chongqing, China; ^2^Department of Gastroenterology, The First Affiliated Hospital of Chongqing Medical University, Chongqing, China

**Keywords:** vitiligo melanocyte, 850K, MDEGs, PPI, functional epigenetic modules

## Abstract

Vitiligo is an pigmentation disorder caused by a variety of pathogenic factors; its main pathophysiological conditions include oxidative stress, immune activation, and genetic background. Additionally, DNA methylation is often associated with the pathogenesis of vitiligo; however, the underlying mechanism remains unknown. In the present study, we used the Human Methylation 850K BeadChip platform to detect DNA methylation changes in the vitiligo melanocytes. We then integrated the results with the transcriptome data of vitiligo melanocytes and lesions to analyse the correlation between differentially methylated levels and differentially expressed genes. The results showed that there was a significant negative correlation between methylation levels and differentially expressed genes. Subsequently, we enriched GO and KEGG based on methylated differentially expressed genes (MDEGs) using R package ClusterProfiler, and the results were closely related to the pathogenesis of vitiligo. In addition, we also constructed a PPI network of MDEGs and excavated three important functional epigenetic modules, involving a total of 12 (BCL2L1, CDK1, ECT2, HELLS, HSP90AA1, KIF23, MC1R, MLANA, PBK, PTGS2, SOX10, and TYRP1) genes. These genes affect melanocyte melanogenesis, cellular oxidative stress and other important biological processes. Our comprehensive analysis results support the significant contribution of the status of DNA methylation modification to vitiligo, which will help us to better understand the molecular mechanism of vitiligo and explore new therapeutic strategies.

## Introduction

Vitiligo is an acquired chronic skin pigmentation disease that affects 0.5–2% of the world's population (1). The reported epidemiological data varies according to region, and the disease impact may be related to the respective social and cultural stigmas (2). The pathogenesis of vitiligo manifests as oxidative stress, infiltration of inflammatory mediators, melanocyte loss, and autoimmune responses (3). It is caused due to the complex interaction among environmental and genetic factors that eventually leads to melanocyte dysfunction (4). However, the inherent defect in melanocytes is an early event in vitiligo; the gradual disappearance of melanocytes may involve multiple processes, including immune system attack, cell degeneration, and exfoliation among others (1).

Epigenetic factors such as DNA methylation, histone modification, and gene silencing through microRNAs play an important role in the pathogenesis and progression of autoimmune dermatoses, such as psoriasis and atopic dermatitis (5). Increasing number of studies have shown that failure to maintain the level and pattern of DNA methylation can lead to abnormal cell function and proliferative activity (6). Although epigenetics plays a key role in the pathogenesis of skin tumors, researchers have recently begun to pay more attention to the epigenetics in the pathogenesis of inflammatory skin diseases, such as psoriasis, atopic dermatitis, and other inflammatory skin diseases (7–9). Abnormal DNA methylation has been identified as a major epigenetic change leading to the development of various skin cancers (10). However, DNA methylation is reversible and is therefore considered an attractive therapeutic intervention (11, 12) to provide the possibility treatment methods for various skin diseases.

Evidences have shown that abnormal DNA methylation is involved in the development of vitiligo. For example, the DNA methylation level of peripheral blood mononuclear cells (PBMC) in vitiligo have been investigated and the emerging evidence suggests an epigenetic regulation of CD8+ T cells. Zhao M et al. reported that in vitiligo patients, DNA methylation of peripheral blood mononuclear cells affects the mRNA expression level of DNMT, MBD, IL-10, and other genes (13). Another study suggests that in autoimmune diseases such as type 1 diabetes (T1D), systemic lupus erythematosus (SLE), and vitiligo genes associated with the proliferation or activation of CD8+ T cells are affected by epigenetic modification (14). However, the important biological pathway regulated by abnormal methylation has not been elucidated yet, and the role of epigenetic factors in the vitiligo pathogenesis needs further research.

**Figure 1:**
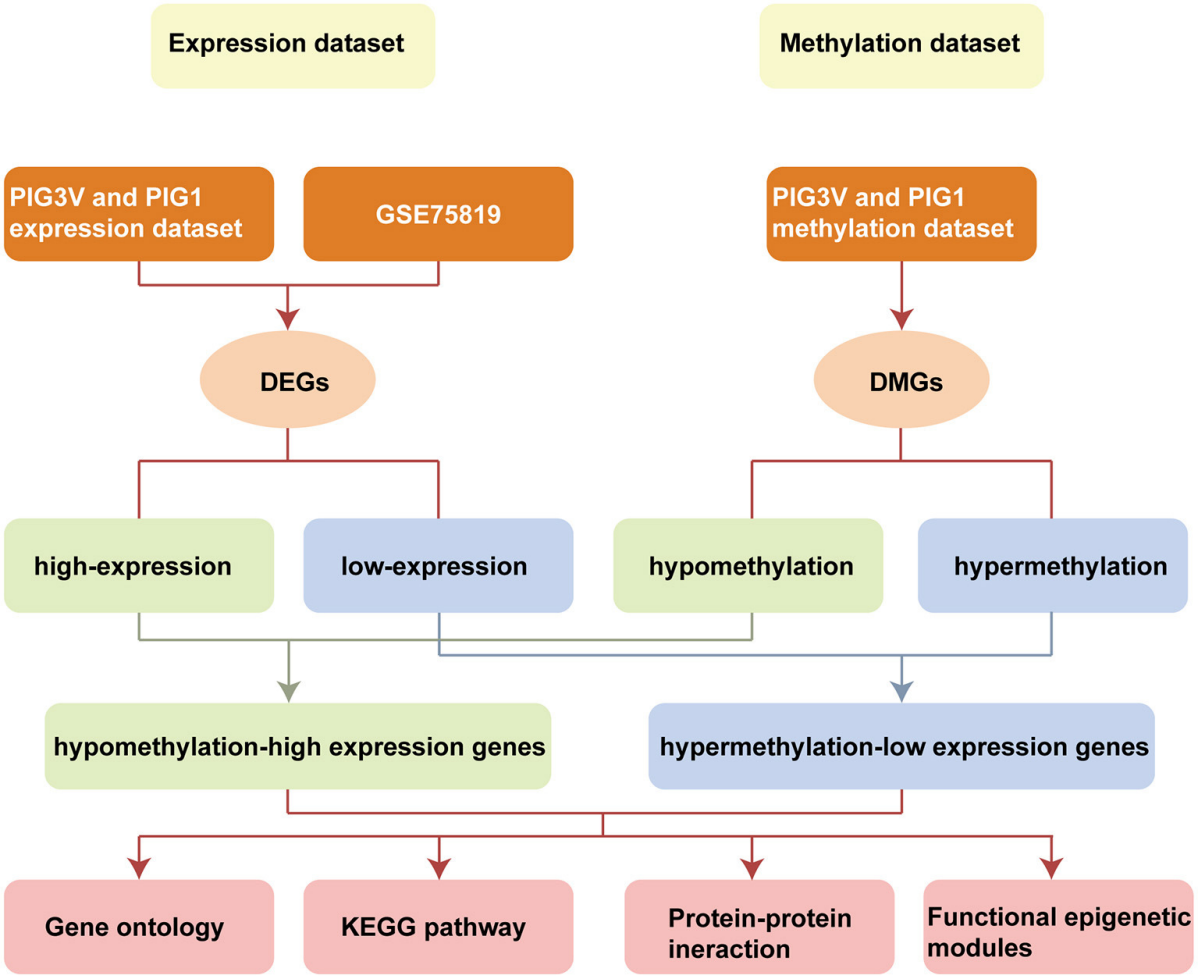
Flowchart of bioinformatics analysis. DMGs, Differentially methylated genes; DEGs, Differentially expressed genes; PIG1, human normal melanocyte; PIG3V, human vitiligo melanocyte.

In the present study, we integrated and analyzed the differential transcriptomic data (from vitiligo melanocyte cell lines and vitiligo lesions) and differential methylation data (from vitiligo melanocyte cell lines). We used Infinium Methylation EPIC BeadChip for the methylation sequencing of vitiligo melanocyte PIG3V and normal melanocyte PIG1. The 850K microarray covered the gene promoter region, gene coding region, CpG island, and enhancer regions present in ENCODE and FANTOM5. The data of the transcriptional group were derived from vitiligo lesion transcriptional sequencing datasets GSE75819 (15 non-segmental vitiligo patients with lesion skin and peri-lesion skin biopsies, the peri-lesion skin biopsies as controls) in the GEO database and our cell line transcriptional sequencing data (PIG3V and PIG1). Subsequently, we conducted GO/KEGG pathway analysis, PPI, and functional epigenetic module analysis of MDEGs to further explore the potential mechanism driven by abnormal methylation of vitiligo. Flowchart of bioinformatics analysis is shown in Figure 1.

## Materials and Methods

### Study Subjects

The human vitiligo melanocyte cell line PIG3V and the human normal melanocyte cell line PIG1 (gifted by Dr. Li Chunying, Xijing Hospital, Fourth Military Medical University, Xi'an, Shanxi Province) were cultured in human melanocyte growth medium 254 supplemented with 5% fetal bovine serum and penicillin- streptomycin at 37°C in a 5% CO2 incubator. To integrate and study the correlation between the methylation status and transcriptome dataset, we sequenced the whole genome of two cases of PIG1 cell line and two cases of PIG3V cell line, and used it as cell line methylation dataset and expression dataset. Vitiligo lesion expression GSE75819 was downloaded from the Gene Expression Omnibus (GEO, https://www.ncbi.nlm.nih.gov/geo/). Fifteen vitiligo focal skin samples and fifteen vitiligo peri-focal skin samples were included in the GSE75819 to verify the expression dataset.

### Infinium Human Methylation EPIC Array Using 850K BeadChip and Data Analysis

DNA was isolated from cell samples, using DNeasy Blood & Tissue Kit (Qiagen, Germany). The purity and concentration of DNA was estimated using Nanodrop 2000 (Thermo Fisher Scientific, China). Approximately 500 ng of genomic DNA from each sample was used for sodium bisulfite treatment using the EZ DNA methylation Gold Kit (Zymo Research, USA) following the manufacturer's protocol. Genome-wide DNA methylation was assessed using the Infinium Human Methylation 850K BeadChip (Illumina Inc, USA) according to the manufacturer's instructions. The array data (IDAT files) were analyzed using the ChAMP package in R for deriving the level of methylation. The methylation status of all the probes was denoted as β value, which is the ratio of the methylated probe intensity to the overall probe intensity (sum of methylated and unmethylated probe intensities plus constant α, where α = 100). A CpG site with |Δβ| ≥ 0.20 (in test vs. control) and adjusted *P*-value ≤ 0.05 was considered as a differentially methylated site. A CpG was considered hypermethylated if Δβ ≥ 0.20 or hypomethylated if Δβ ≤ −0.20. The average β value of promoters and CpG islands was compared between diseased and normal conditions. Promoters and CGIs with |Δβ| ≥ 0.20 and adjusted *P*-value ≤ 0.05 were considered for further analysis.

### Copy Number Variation Analysis

We used the consumer package to analyse the human measurement epic data to identify copy number variation (CNV). CNV analysis was carried out in two steps. First, the combined signal values of “methylated” and “unmethylated” CPGs were normalized using the control group (PIG1 as control). This step required correcting the probe and sample deviation. Second, the adjacent probes were combined to generate a probe bin with the minimum size and quantity to reduce the remaining technical changes and achieve meaningful segmentation results. For CNV results and differential methylation sites, we used the Circos map for joint analysis.

### Acquisition and Processing of Expression Spectrum Data

RNA-Seq strand-specific libraries were constructed using a VAHTS Total RNA-Seq (H/M/R) Library Prep Kit (Vazyme, China). The original gene expression data set GSE75819 was downloaded from the GEO public database. We used R 4.0.0 to analyse the expression matrix. The robust multiarray average (RMA) method was used to pre-process data, including background adjustment, normalization, and log value conversion. The Limma package was used to search for differential genes. The threshold of up and down genes was set as |log FC| ≥ 1 and *P* ≤ 0.05.

### FunRich

FunRich 3.1.3 (http://www.funrich.org) is an independent software tool, used mainly for the functional enrichment and interaction network analysis of genes and proteins (15). The software was used to identify hypomethylation and hypermethylation genes for further analysis.

### GO and KEGG Enrichment

GO and KEGG enrichment were carried out for hypomethylation and hypermethylation genes using R package clusterProfiler. Results with *P* < 0.05 were considered statistically significant. We used R package ggplot2 to visualize the important projects of each group function and pathway enrichment analysis.

### Construction of PPI Network and Related Analysis

To further explain the interactions between the two sets of genes in the pathogenesis of vitiligo and the specific molecular mechanisms, PPI analysis was performed. Since the number of hypomethylation and hypermethylation genes was small, we integrated them to construct PPI networks. STRING 11.0 was used to generate a PPI network (https://string-db.org/). The cutoff value was set to 0.4 of the interaction score. The results were imported into Cytoscape 3.8.0 for subsequent analysis. CytoHubba app (http://apps.cytoscape.org/apps/cytohubba) was used to screen the hub genes. The first 3 modules were applied in Cytoscape by the MCODE app software package. We used FunRich 3.1.3 to identify and visualize the potential genes related to each module.

### Statistical Analysis

The correlation between differentially expressed genes and methylation levels was analyzed by Pearson's correlation coefficient. Results with *P* < 0.05 were considered statistically significant. The statistical analyses were performed using R 4.0.0. GraphPad Prism 8 (GraphPad Prism Software Inc., San Diego, California) to correlate analysis and graphic display.

## Results

### Global Methylation Pattern of Vitiligo Melanocytes and Normal Melanocytes

**Figure 2:**
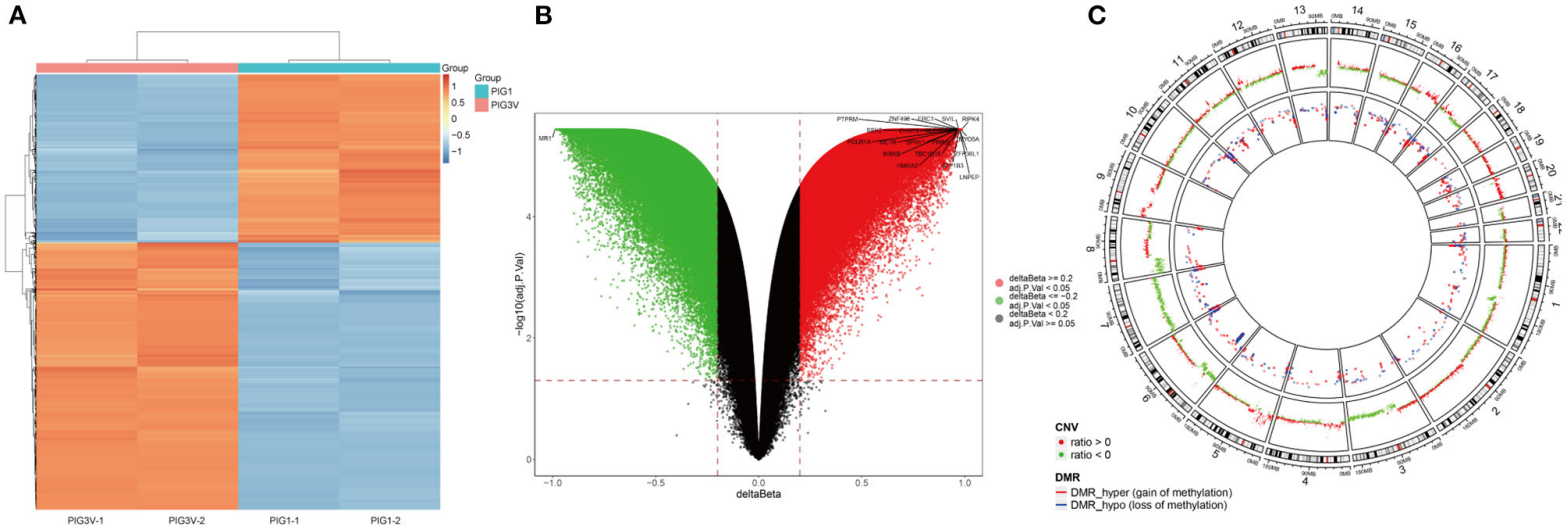
**(A)** The heat map, **(B)** the Volcano map analysis of genes with differential methylation between vitiligo melanocyte and normal melanocyte. **(C)** A joint analysis of CNV results and differential methylation sites performde by the Circos diagram. CNV and the outermost circle represent chromosome regions, and the second inward circle represents the CNV copy number (red represents ratio > 0, green represents ratio < 0), The inner circle represents the DMR area.

The characteristics of DNA methylation were significantly different between vitiligo melanocyte cell line and normal melanocyte cell line. The analysis of differential methylation position (DMP) showed 83 451 differential methylation positions in PIG3V compared with PIG1. Among them, 53 895 were hypermethylated and 29 556 were hypomethylated. CpGs showed bimodal methylation distribution in PIG3V, and most of them showed hyper/hypo methylation levels. The differential methylation positions are shown in the cluster heat map (Figure 2A) and the volcano map (Figure 2B). The genes corresponding to the top 20 differences in methylation positions are listed at the two tips of the volcanic map. In addition, to examine the methylation changes throughout the genome, we used Circos maps for joint analysis (Figure 2C).

### Integration of Epigenomic and the Transcriptomic Data

**Figure 3:**
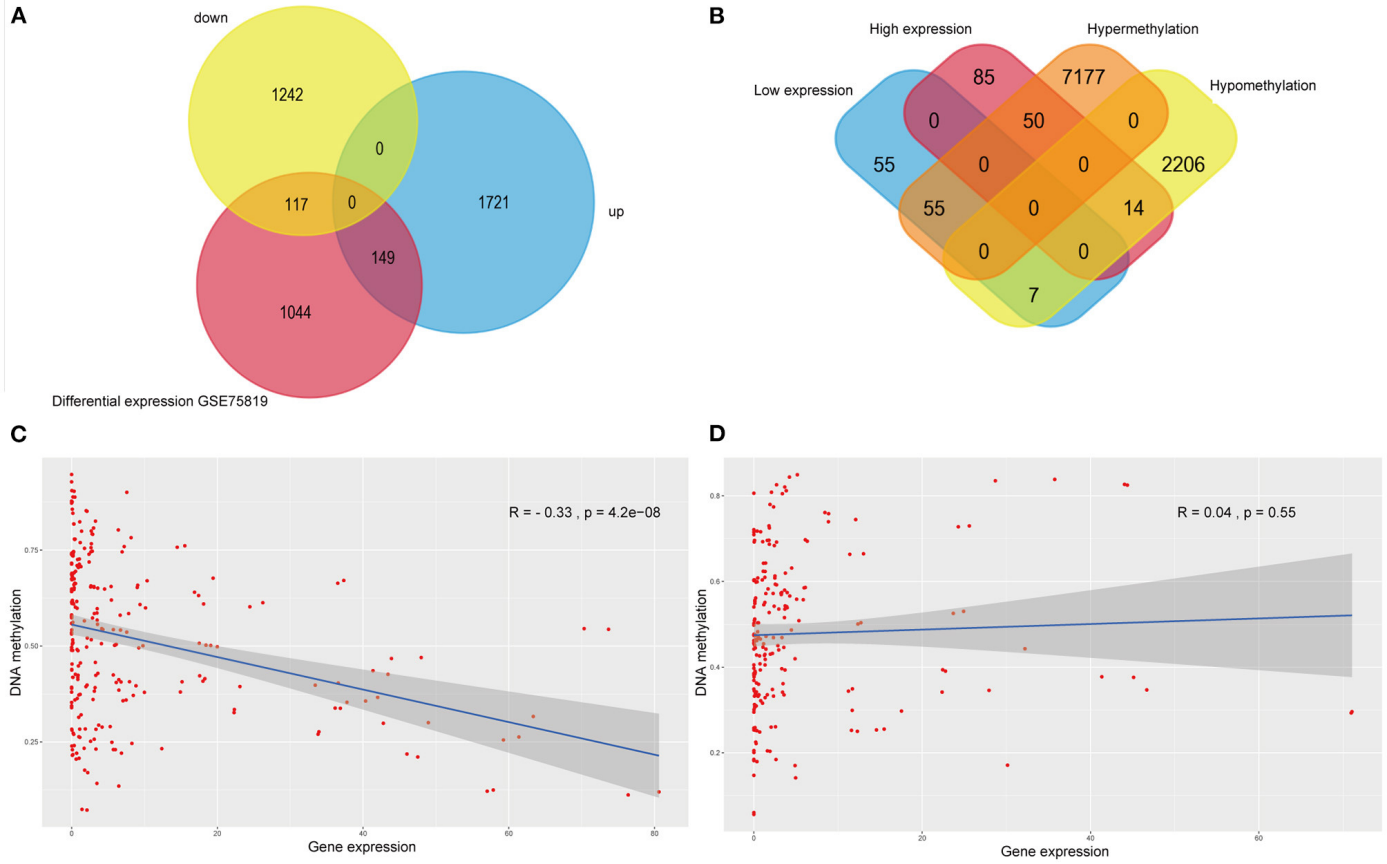
Integrative analysis of methylome and transcriptome of vitiligo. **(A)** Veen diagram of differentially expressed genes between vitiligo skin lesion microarray and vitiligo melanocyte microarray. **(B)** Venn diagram between differentially methylated genes and differentially expressed genes of vitiligo. Correlation analysis between differentially methylated genes and differentially expressed genes: **(C)** negative correlation analysis, **(D)** positive correlation analysis.

The intersection of transcriptomic data on vitiligo cell lines and lesions was visualized in the veen map (Figure 3A); there were 117 down-regulated and 149 up-regulated genes. The intersection of transcriptomic methylation data of vitiligo melanocyte cell line was also visualized by veen map (Figure 3B) and 126 overlapping genes were obtained. We analyzed the correlation between intersectional genes and their methylation levels, and the results showed that DNA methylation in vitiligo melanocyte cell line is negatively correlated with gene expression (Figure 3C), but there was no significant positive correlation (Figure 3D). Therefore, we selected the genes with a high expression of low methylation and low expression of high methylation for further study. There were 69 genes that met these requirements, including 14 down-regulated genes with hypermethylation and 55 up-regulated genes with low methylation. It is important to note that GEO currently does not have DNA methylation data for vitiligo, so there is no verification set for vitiligo methylation.

### Enrichment Analysis of GO and KEGG Pathways

**Figure 4:**
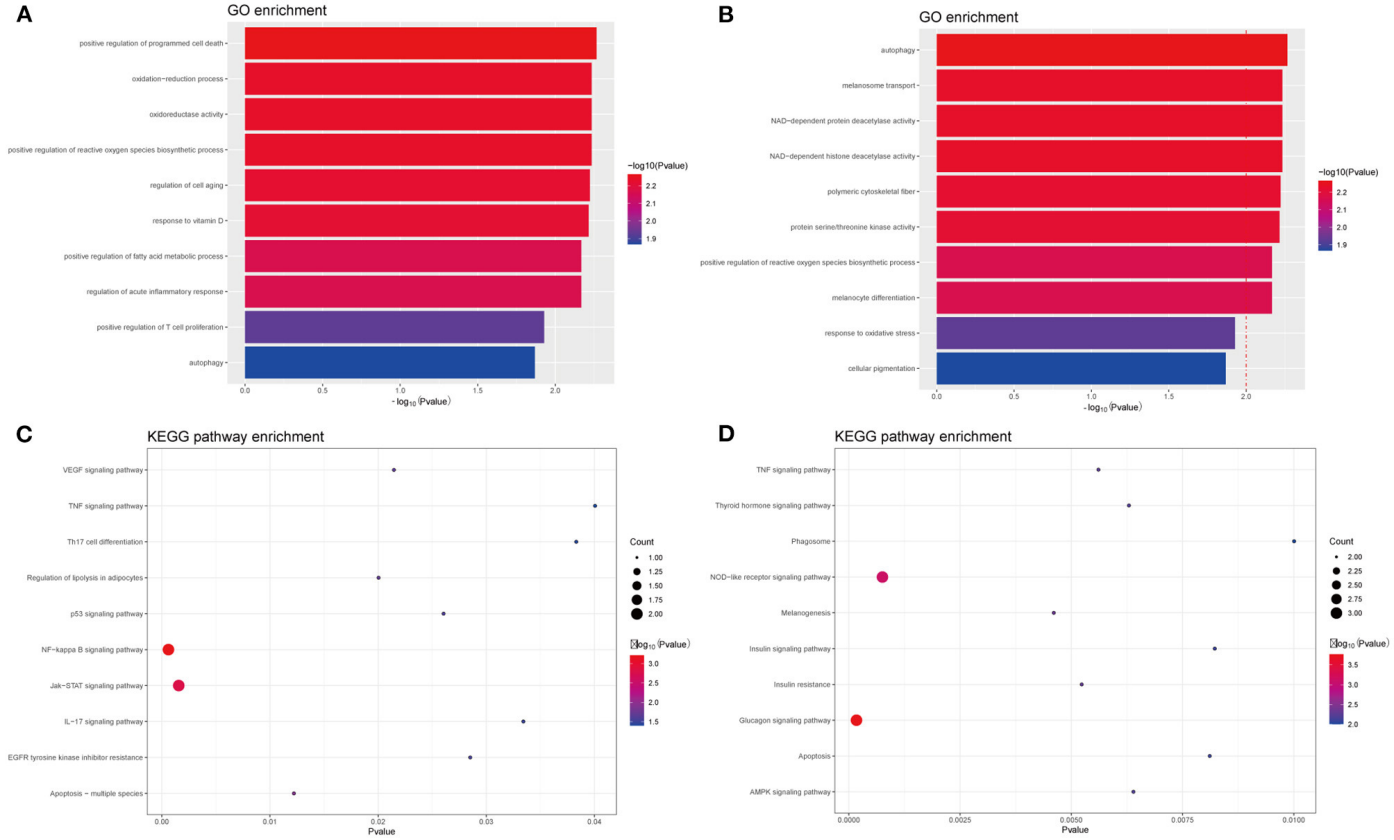
GO and KEGG enrichment analysis of methylated-differentially expressed genes related with vitiligo melanocyte. Top 10 GO terms in **(A)** GO enrichment analysis of Hyper-MDGEs, **(B)** GO enrichment analysis of Hypo-MDGEs. **(C)** KEGG pathway analysis of Hyper-MDGEs, **(D)** KEGG pathway analysis of Hypo-MDGEs. The horizontal axes shows -log10 transformed *P*-value and *p* < 0.05 is considered significant.

In PIG3V, the biological processes of MDEGs, such as oxidation–reduction oxidoreductase activity, are mainly related to oxidative stress, immune responses, and melanogenesis (Figures 4A,B). The response to oxidative stress is related to the positive regulation of T cell proliferation, whereas regulation of acute inflammatory responses is involved in inflammation, and melanocyte differentiation and melanosome transport affect melanogenesis. The specific genes involved in each GO term are shown in the Supplementary Table 1. Apart from these, some other processes such as the regulation of cell aging, response to vitamin D, autophagy, and the positive regulation of fatty acid metabolic processes are related to the pathogenesis of vitiligo.

KEGG analysis identified a variety of pathways in enrichment analysis, and we visualized the top 10 (Figures 4C,D). According to KEGG classification, the pathways rich in MDEGs are commonly involved in IL-17 signaling, Th17 cell differentiation, TNF signaling, and NOD-like receptor signaling pathway. Further, different pathways may overlap due to common elements. For the transcripts of differential regulation, some pathways are concentrated in the glucagon signaling such as insulin resistance, insulin signaling, and thyroid hormone signaling. This may be related to the crosstalk among vitiligo, diabetes, thyroid, and other autoimmune diseases, and the MDEGs involved in these pathways may be important biomarkers for the mutual crosstalk in immune diseases.

### PPI Network and Functional Epigenetic Modules

**Figure 5:**
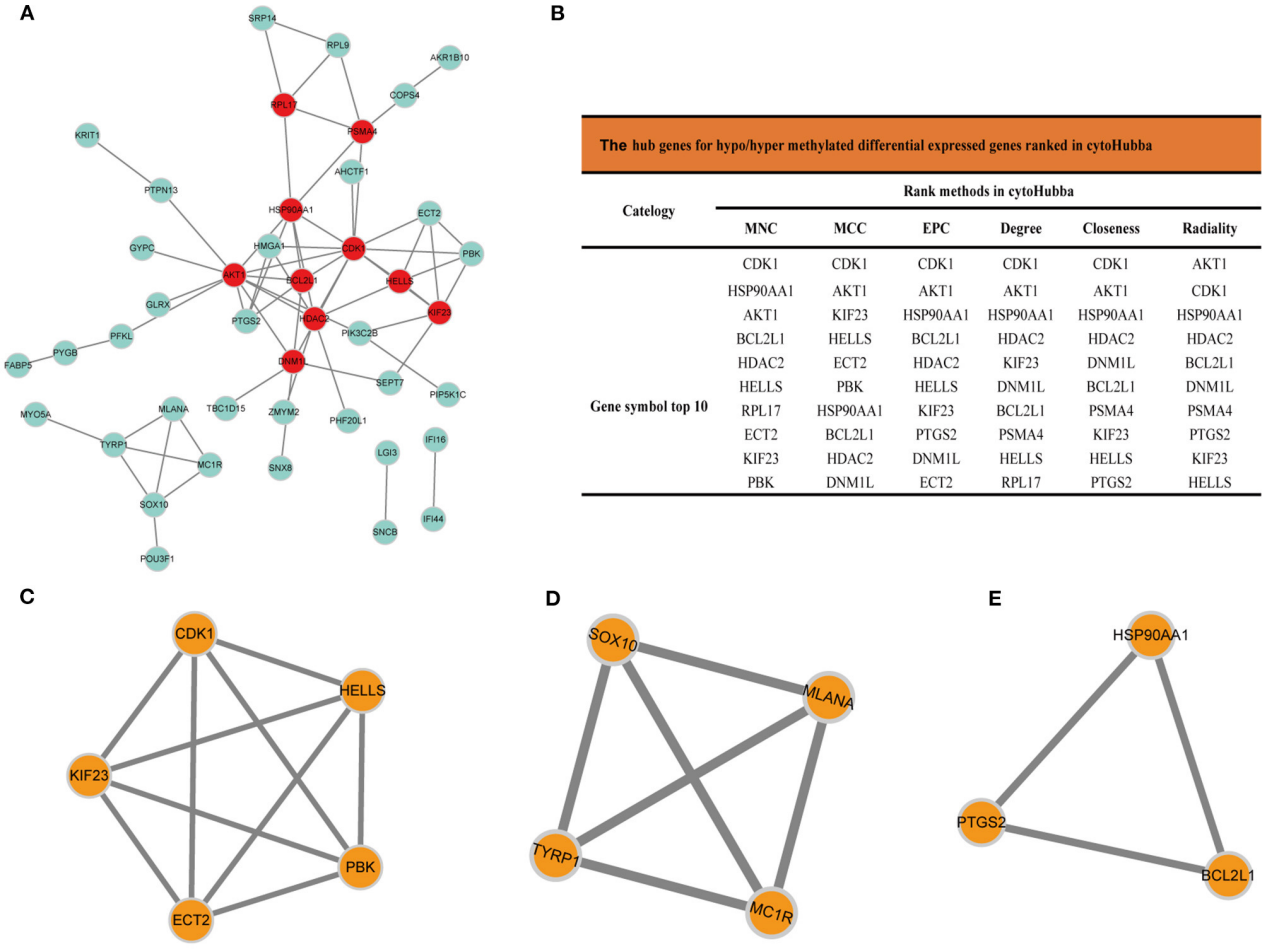
**(A)** Protein-protein interaction network of MDEGs, Disconnected nodes were hid in the network. **(B)** Hub genes for MDEGs ranked in cytoHubba, functional epigenetic modules of the protein-protein interaction network: **(C)** module1, **(D)** module2, **(E)** module3.

The PPI network analyzed by STRING showed 69 nodes and 66 edges. Cytoscape 3.8.0 was used to visualize the PPI network (Figure 5A), CytoHubba app in Cytoscape software is used to select the hub nodes genes of the PPI network. In the results, a total of 7 genes of the top 10 hub genes detected by six ranked methods in cytoHubba were overlap hub genes (Figure 5B), including CDK1 (cyclin dependent kinase 1), HSP90AA1 (heat shock protein 90 alpha family class A member 1), AKT1 (AKT serine/threonine kinase 1), BCL2L1 (BCL2 like 1), HDAC2 (histone deacetylase 2), HELLS (helicase, lymphoid specific), and KIF23 (kinesin family member 23). Among them, BCL2L1 was hypermethylated and its expression was downregulated, whereas the other six genes are hypomethylated and their expression was upregulated. Finally, based on the PPI network, three important epigenetic function modules were identified and were established as statistically significant. The results of enrichment analysis of functional epigenetic modules showed that the MDEGs of module 1 (Figure 5C) played an important role in the regulation of cell cycle and cell division, and the MDEGs of module 2 (Figure 5D) affected melanogenesis and pigmentation. Module 3 (Figure 5E) involved the regulation of cellular response to stress, negative regulation of intrinsic apoptotic signaling pathway, and complex pathway regulation. The enrichment results are presented in detail in Table 1.

**Table 1:** GO and KEGG enrichment analysis of functional epigenetic modules related with vitiligo melanocyte.

**Category**	**Term**	**Count**	**FDR**	**Gene**
Module1	GO:0072686 → mitotic spindle	3	4.33E-05	CDK1, ECT2, KIF23
	GO:0097149 → centralspindlin complex	2	4.33E-05	ECT2, KIF23
	GO:0030496 → midbody	3	0.00017	CDK1, ECT2, KIF23
	GO:0005524 → ATP binding	4	0.0097	CDK1, HELLS, KIF23, PBK
	GO:0004674 → protein serine/threonine kinase activity	2	0.0233	CDK1, PBK
	GO:0007049 → cell cycle	5	0.00072	CDK1, ECT2, HELLS, KIF23, PBK
	GO:0051301 → cell division	4	0.00072	CDK1, ECT2, HELLS, KIF23
	GO:0000278 → mitotic cell cycle	4	0.0011	CDK1, ECT2, KIF23, PBK
	GO:0090068 → positive regulation of cell cycle process	3	0.0043	CDK1, ECT2, KIF23
	GO:0032467 → positive regulation of cytokinesis	2	0.0052	ECT2, KIF23
	GO:0042307 → positive regulation of protein import into nucleus	2	0.0052	CDK1, ECT2
	GO:0000281 → mitotic cytokinesis	2	0.0055	ECT2, KIF23
	GO:0070301 → cellular response to hydrogen peroxide	2	0.0071	CDK1, ECT2
	GO:1903047 → mitotic cell cycle process	3	0.0082	CDK1, ECT2, KIF23
	GO:0071478 → cellular response to radiation	2	0.0144	ECT2, PBK
	GO:0006323 → DNA packaging	2	0.019	CDK1, HELLS
	GO:0022607 → cellular component assembly	4	0.019	CDK1, ECT2, HELLS, KIF23
	GO:0051276 → chromosome organization	3	0.0218	CDK1, HELLS, KIF23
	GO:0010038 → response to metal ion	2	0.0395	CDK1, ECT2
	GO:0001932 → regulation of protein phosphorylation	3	0.0405	CDK1, ECT2, PBK
	GO:0032147 → activation of protein kinase activity	2	0.0405	CDK1, ECT2
	GO:0016569 → covalent chromatin modification	2	0.0411	CDK1, HELLS
	GO:0000226 → microtubule cytoskeleton organization	2	0.0459	CDK1, KIF23
	GO:0065003 → protein-containing complex assembly	3	0.0468	CDK1, ECT2, HELLS
Module2	GO:0042470 → melanosome	2	0.0141	MLANA, TYRP1
	GO:0043473 → pigmentation	2	0.0332	MC1R, TYRP1
	hsa04916 → Melanogenesis	2	0.00062	MC1R, TYRP1
Module3	GO:0042803 → protein homodimerization activity	3	0.0057	BCL2L1, HSP90AA1, PTGS2
	GO:0019899 → enzyme binding	3	0.0263	BCL2L1, HSP90AA1, PTGS2
	GO:0019901 → protein kinase binding	2	0.041	BCL2L1, HSP90AA1
	GO:0019904 → protein domain specific binding	2	0.0436	BCL2L1, HSP90AA1
	GO:0007006 → mitochondrial membrane organization	2	0.0114	BCL2L1, HSP90AA1
	GO:0009408 → response to heat	2	0.0114	HSP90AA1, PTGS2
	GO:0009628 → response to abiotic stimulus	3	0.0114	BCL2L1, HSP90AA1, PTGS2
	GO:0019221 → cytokine-mediated signaling pathway	3	0.0114	BCL2L1, HSP90AA1, PTGS2
	GO:0033138 → positive regulation of peptidyl-serine phosphorylation	2	0.0114	HSP90AA1, PTGS2
	GO:0045429 → positive regulation of nitric oxide biosynthetic process	2	0.0114	HSP90AA1, PTGS2
	GO:0051726 → regulation of cell cycle	3	0.0114	BCL2L1, HSP90AA1, PTGS2
	GO:0071478 → cellular response to radiation	2	0.0114	BCL2L1, PTGS2
	GO:0080135 → regulation of cellular response to stress	3	0.0114	BCL2L1, HSP90AA1, PTGS2
	GO:1903827 → regulation of cellular protein localization	3	0.0114	BCL2L1, HSP90AA1, PTGS2
	GO:1904407 → positive regulation of nitric oxide metabolic process	2	0.0114	HSP90AA1, PTGS2
	GO:2001243 → negative regulation of intrinsic apoptotic signaling pathway	2	0.0114	BCL2L1, PTGS2
	GO:0006839 → mitochondrial transport	2	0.0122	BCL2L1, HSP90AA1
	GO:0010647 → positive regulation of cell communication	3	0.0174	BCL2L1, HSP90AA1, PTGS2
	GO:0023056 → positive regulation of signaling	3	0.0174	BCL2L1, HSP90AA1, PTGS2
	GO:0046677 → response to antibiotic	2	0.0185	BCL2L1, HSP90AA1
	GO:1902531 → regulation of intracellular signal transduction	3	0.0185	BCL2L1, HSP90AA1, PTGS2
	GO:0001101 → response to acid chemical	2	0.0196	BCL2L1, PTGS2
	GO:0009653 → anatomical structure morphogenesis	3	0.0247	BCL2L1, HSP90AA1, PTGS2
	GO:0048584 → positive regulation of response to stimulus	3	0.0263	BCL2L1, HSP90AA1, PTGS2
	GO:0048608 → reproductive structure development	2	0.0275	BCL2L1, PTGS2
	GO:0009636 → response to toxic substance	2	0.0316	BCL2L1, PTGS2
	GO:0032990 → cell part morphogenesis	2	0.0316	BCL2L1, HSP90AA1
	GO:0051186 → cofactor metabolic process	2	0.0316	HSP90AA1, PTGS2
	GO:0071417 → cellular response to organonitrogen compound	2	0.0319	BCL2L1, PTGS2
	GO:0006897 → endocytosis	2	0.0345	BCL2L1, HSP90AA1
	GO:0045786 → negative regulation of cell cycle	2	0.0345	BCL2L1, PTGS2
	GO:1903047 → mitotic cell cycle process	2	0.0389	BCL2L1, HSP90AA1
	GO:0007276 → gamete generation	2	0.0421	BCL2L1, PTGS2
	GO:0007346 → regulation of mitotic cell cycle	2	0.0421	BCL2L1, HSP90AA1
	GO:0043065 → positive regulation of apoptotic process	2	0.0421	BCL2L1, PTGS2
	GO:0010564 → regulation of cell cycle process	2	0.0464	BCL2L1, HSP90AA1
	hsa05200 → Pathways in cancer	3	0.00079	BCL2L1, HSP90AA1, PTGS2
	hsa04064 → NF-kappa B signaling pathway	2	0.0015	BCL2L1, PTGS2
	hsa04657 → IL-17 signaling pathway	2	0.0015	HSP90AA1, PTGS2
	hsa05222 → Small cell lung cancer	2	0.0015	BCL2L1, PTGS2
	hsa04621 → NOD-like receptor signaling pathway	2	0.0019	BCL2L1, HSP90AA1
	hsa04151 → PI3K-Akt signaling pathway	2	0.0068	BCL2L1, HSP90AA1

## Discussion

Vitiligo is considered to be an autoimmune disease because cytokines such as (IFN)-γ, IL-1, IL-6, IL-8, and IL-10 are overexpressed in lesions, and activated CD8 + T lymphocytes, TH17, and other immune cells are significantly aggregated in the lesion area (16–19). However, some researchers have suggested that autoimmunity may be secondary to high oxidative stress in vitiligo skin, leading to inherent defects of melanocytes and changes in its microenvironment (4). At present, the unified view is that the inherent defect of melanocytes is an early event in vitiligo, and the gradual disappearance of epidermal melanocytes leads to skin depigmentation of vitiligo lesions.

GO enrichment analyses of the oxidation-reduction process, response to oxidative stress, and oxidoreductase activity have been partially studied in vitiligo (20–23). Inflammation-related processes such as positive regulation of T cell proliferation, regulation of acute inflammatory response, and melanogenesis-related processes such as cellular pigmentation, melanocyte differentiation, melanosome transport were also widely studied in vitiligo (24–26). However, previous studied did not consider methylation modification, and our study shows that the methylation levels of key genes involved in these important regulatory processes, such as TYP1, IL17, and MC1R, are altered in vitiligo melanocyte PIG3V. Therefore, based on our findings, we propose that the change in methylation level may regulate the differential expression of functional genes in vitiligo. To the best of our knowledge, this is the first study to analyze the methylation profile of vitiligo melanocytes.

The infiltration of immune cells and the release of pro-and anti-inflammatory cytokines is key to the pathogenesis of vitiligo. KEGG results showed that MDEGs were significantly enriched in IL-17 signaling pathway, Th17 cell differentiation, TNF signaling pathway, and NOD-like receptor signaling pathways, and these signaling pathways have been proved to be involved in mediating the immune regulation of vitiligo (18, 27, 28). In addition, differential methylation of AKT1, PYGB, HDAC2 were frequently observed in the insulin signaling and thyroid hormone signaling pathways. These pathways play an important role in abnormal glucose metabolism in diabetes and thyroid diseases (29–31), while vitiligo is frequently associated with other autoimmune diseases, particularly autoimmune thyroid diseases (Hashimoto's thyroiditis and Graves' disease), adult-onset type 1 diabetes mellitus, psoriasis, and so on (32). Thus, genes in these signaling pathways are regulated by methylation modification may play an important role in crosstalk between immune diseases. Previous studies have shown that the effective treatment of psoriasis, an immune inflammatory skin disease, can reverse DNA methylation in the epidermis (33, 34), suggesting that further studies on vitiligo methylation may be potentially identify new treatment strategies for vitiligo.

Futhermore, the PPI network showed that CDK1, HSP90AA1, AKT1, BCL2L1, HDAC2, HELLS, and KIF23 are 7 central genes identified by the top 10 high hub nodes after gene overlap. Among these central genes, CDK1 is a set of Ser/Thr kinase system corresponding to cell cycle progression (35), and its differential expression was found in another vitiligo study (36); HSP90AA1 plays a key role in signal transduction, protein folding, protein degradation, and morphological evolution (37); AKT1 and BCL2L1 are involved in apoptosis (38, 39), and AKT methylation has been shown to promote skin tumors (40); HDAC2 interferes with histone deacetylase function (41); KIF23 plays an important role in the changes of cellular motor cytoskeleton (42); HELLS is considered to be a regulator of cell senescence and is necessary for DNMT1-mediated methylation maintenance and DNMT3A/DNMT3B-mediated remethylation (43). Therefore, we speculate that these hub genes may be involved in the pathological process in the early stage of melanocyte loss in vitiligo, but further functional experiments are needed to further explore.

The analysis of the epigenetic module shows that the MDEGs in module 1 is highly enriched in the processes of cell cycle regulation, whereas the MDEGs in module 2 is involved in the process of pigmentation and the pathway of regulating melanin production. Interestingly, the functions of MDEGs in these modules are relatively concentrated, which suggests that these biological processes may be collectively regulated by methylation. Whether demethylation can reverse these processes needs to be verified by further studies. Module 3 involves cellular stress, apoptosis, and regulation of complex biological processes and pathways (44, 45) key to the pathogenesis of vitiligo. These findings suggest that MDEGs in vitiligo melanocytes may have regulatory functions in these biological processes and molecular functions. However, some genes and pathways identified in this study have not been formally studied as targets of the vitiligo process, and need to be evaluated further.

Although our research attempts to bridge an important gap, it does have several limitations that can be addressed in the future studies. Firstly, the sample size was relatively small as only four microarray profiles were analyzed and there was no vitiligo methylation data available on the GEO platform. Hence, a replication with larger samples will be required to validate the findings. Secondly, the study lacked experimental verification of the effects of aberrant methylation on gene expression and functions in vitiligo melanocytes. Therefore, supplementary molecular experiments should be conducted to verify the results of our investigation. Furthermore, due to the limited sample size, the GEO verification set of differentially expressed genes selected only differential datasets rather than general high and low expression sets due to the differences between cell lines and human samples. This strict intersection may mask genes that play significant roles.

In conclusion, using a series of bioinformatics databases and tools, we found that the interactions among MDEGs with different functions and signaling pathways is related to the pathogenesis of vitiligo melanocytes. The hub genes of vitiligo melanocytes include CDK1, HSP90AA1, AKT1, BCL2L1, HDAC2, HELLS, and KIF23. The genes involved in three important epigenetic modules include BCL2L1, CDK1, ECT2, HELLS, HSP90AA1, KIF23, MC1R, MLANA, PBK, PTGS2, SOX10, and TYRP1. This study provides hypothetical and biological characteristic insight into the pathogenesis of vitiligo. However, additional molecular-level studies are needed to confirm the identified genes and pathways in vitiligo to elucidate potential mechanisms, and *in vitro* and *in vivo* functional studies are also required to find the crucial role of the identified genes in vitiligo pathogenesis.

## Data Availability Statement

The raw data supporting the conclusions of this article will be made available by the authors, without undue reservation.

## Author Contributions

YP and XC prepared the figures and writing. YC and LZ collected and organized the data. JiaC cultured cells. YZ and XS prepared the tables. JinC critically revised the data. All authors contributed to the article and approved the submitted version.

## Conflict of Interest

The authors declare that the research was conducted in the absence of any commercial or financial relationships that could be construed as a potential conflict of interest.
